# Monitoring of Bridges by a Laser Pointer: Dynamic Measurement of Support Rotations and Elastic Line Displacements: Methodology and First Test

**DOI:** 10.3390/s18020338

**Published:** 2018-01-25

**Authors:** Serena Artese, Vladimiro Achilli, Raffaele Zinno

**Affiliations:** 1 Department of Civil Engineering, University of Calabria, Via Bucci cubo 45B, 87036 Rende, Italy; 2 Department of Civil, Environmental and Architectural Engineering, Via Marzolo, 9, 35131 Padova, Italy; vladimiro.achilli@unipd.it; 3 Department of Informatics, Modeling, Electronic and System Engineering, University of Calabria, Via P. Bucci cubo 42C, 87036 Rende, Italy; raffaele.zinno@unical.it

**Keywords:** laser pointer, displacement monitoring, laser fingerprint, video, data synchronization

## Abstract

Deck inclination and vertical displacements are among the most important technical parameters to evaluate the health status of a bridge and to verify its bearing capacity. Several methods, both conventional and innovative, are used for structural rotations and displacement monitoring; however, none of these allow, at the same time, precision, automation, static and dynamic monitoring without using high cost instrumentation. The proposed system uses a common laser pointer and image processing. The elastic line inclination is measured by analyzing the single frames of an HD video of the laser beam imprint projected on a flat target. For the image processing, a code was developed in Matlab^®^ that provides instantaneous rotation and displacement of a bridge, charged by a mobile load. An important feature is the synchronization of the load positioning, obtained by a GNSS receiver or by a video. After the calibration procedures, a test was carried out during the movements of a heavy truck maneuvering on a bridge. Data acquisition synchronization allowed us to relate the position of the truck on the deck to inclination and displacements. The inclination of the elastic line at the support was obtained with a precision of 0.01 mrad. The results demonstrate the suitability of the method for dynamic load tests, and the control and monitoring of bridges.

## 1. Introduction

The possibility to perform fast and accurate image processing, thanks to the power of the most recent computers, allows us to conceive new exciting applications of this technology in several fields and, in particular, for the monitoring of large structures. The projection of the tangent to the elastic line of a girder, materialized by a light beam, on an image sensor or on a target, can be effectively used for this purpose. Nowadays this is possible in a cheap and simple way thanks to laser technology. Several laser pointers, characterized by a low cost, small dimensions and weight, low power, limited beam divergence, and good pointing stability, are presently available on the market. All these characteristics allow the development of a technique for monitoring large structures, and in particular, bridges.

Inclinations and vertical displacements are among the most relevant technical parameters for assessing the health status of a bridge structure and for checking its load capacity. These parameters are also used to verify if the structural response of a bridge under various loading conditions is the one foreseen in the design phase. To control the state of health of a bridge before the opening to traffic, the structure is usually charged by static loads, materialized by a convoy of heavy trucks parked on the deck in known positions. The deflections of the girders are then measured by using levels or total stations, while rotations are in general obtained by inclinometers. Static tests, in relation to the importance of the work, are supplemented by dynamic tests on structural elements [1]. Load tests are performed by following similar procedures in several countries [2,3,4,5].

For Structural Health Monitoring (SHM), and in particular for bridges, several physical and mechanical parameters are measured. For this aim, Fiber Optic Sensors (FOS) are very useful, thanks to their capability to monitor different measurands. The problem of FOS is the need to assure that the sensor system itself is not damaged either when deployed in the field or during the working life. For that reason, it is often necessary to monitor the sensors them-selves [6].

The use of structural vibration data is increasingly widespread; in this field, we can recognize four main methods, based on: (a) natural frequency, (b) mode shape, (c) curvature/strain mode shape, and (d) other modal parameters; a review of vibration-based methods is reported in [7].

Also widespread is the indirect bridge monitoring performed by means of instrumented vehicles, without using sensors on the structure; see [8] and references therein. 

For large structures, wireless sensor networks are more and more diffused [9]. Some applications make use of a few thousands of sensors for a single bridge [10]. 

Several methods, both conventional and innovative, are used for structural movement monitoring; all of these have pros and cons: (1) Dial gauges, often-used for measurements of floor slabs deflections, are difficult to install and manage, due to the height of bridges and the presence of water; (2) Digital levels are characterized by high precision, but they cannot perform dynamic multi-target measurements; (3) Robotic total stations can perform 3D coordinates measurements with a sampling rate of up to 7 Hz and for velocities up to about 10 cm/s [11,12,13]. The high precision and the automation of measurement can be joined to the possibility of data transfer over the internet and remote management [14], but the high cost of these high-end instruments limits their use for long-term bridge monitoring; (4) GNSS satellite-surveying is often used for long span bridges [15,16,17,18]. The attainable precision is high and the maximum sampling rate exceeds 20 Hz for the recent instruments. The main disadvantage is due to the mandatory antenna positioning on the point to measure; (5) Terrestrial laser scanning (TLS) and photogrammetry are by now consolidated techniques both for lab-tests [19] and for the surveying of bridges under static or operating conditions [20,21,22,23]. The comparison of scans acquired at different times allows us to obtain, e.g., the deviations between corresponding points of the bridge surface in different situations (loads, temperature, etc.). With regard to dynamic monitoring, the high sampling rate of line scanners, used in Mobile Mapping Systems, can be exploited. In particular, rotations and deflections of the superstructure of a bridge could be dynamically measured in near real time. One must consider that the best fitting line has in general a better accuracy than that of each single measured point, so the final result could reach a precision higher than that declared for the instrument used [24]; (6) Micro Electro-Mechanical Systems (MEMS) have recently been proposed for deflection measurement using inclination parameter measurements [25]. The results are affected by the high S/N ratio for dynamic tests; (7) Digital Image Correlation is a promising technique for bridge deflection measurements, also thanks to the increasing resolution of the last digital cameras [26,27,28].

The use of a laser beam for measuring deflections is by now a consolidated technique. A laser-based displacement/deflection measurement system is described in [29]. In order to achieve a remote measurement, the laser beam of a digital level is collimated and directed to a detector array, which is attached to the remote object to be measured. The system is not suitable for long-term measurements, since the level and the array must be placed on the monitored object and these expensive instruments must be left unattended. 

Recently, a concept of measuring devices using a laser diode and a CCD camera has been proposed for structural monitoring [30]. 

Among the several technologies used for structures dynamic displacement monitoring, the methods based on laser projection-sensing are increasingly being used, thanks to the availability of low-cost hardware. A system based on multiple laser-vision modules, each composed of a two-point laser, a camera, and a screen such that one module can act as a reference for the neighboring modules, was proposed in [31]. The estimation of relative displacements between two sides was based on an assumption that there is zero initial displacement and that three laser beams are always on the screens. To overcome these limits, a calibration method was proposed afterwards [32]. Another application, using several cameras and targets, was proposed in [33] and improved in [34]. All of these systems use a subsystem for every point to be monitored.

In this context, the methodology described below, able to conjugate a high precision, low cost, and easiness of use has been developed [35]. The measurement of the inclination is obtained by the variation of the tangent to the elastic line, materialized by the laser beam generated by a pointer attached to the deck bridge structure, and projected on a screen located at an adequate distance, in order to amplify the movement of the laser fingerprint and to get, therefore, a remarkably accurate result. A video of the oscillations of the laser footprint during the monitoring activities is acquired. By analyzing the single frames, the variable position of the laser footprint centroid gives information about the inclination changes and, consequently, about the dynamic deflections. The position of the dynamic load can be detected by a video and/or GNSS positioning. The synchronization of acquisitions is performed using GPS time. 

The method is characterized by: (1) Moderate cost; (2) Low weight and small size hardware; (3) Ease of installation; (4) The precision requested for bridge deflection monitoring; and (5) High frame rate (30 frames per second, upgradeable to 120 by using a common action camera).

A method for displacement monitoring using a laser beam, a projection plate plane, and a camera has been presented in [36]. The method described in the present paper differs mainly in the following aspects: (1) The laser device used in [36] must have its optical path direction perpendicular to the movement direction and rotations are not taken into account; (2) The lab tests described in [36], conducted on a bridge model, refer to loads in a fixed position, so the synchronization of load positioning and image capturing, which represents a fundamental topic for dynamic monitoring, is not considered; (3) Our test has been performed on a real bridge, with a real mobile load. 

Another research work [37] has already shown field test results using a laser and a camera. Also in this case, however, there is no connection between load position and rotational measurements. 

In the following, the methodology for the monitoring of dynamic inclination and displacement of a bridge by using a low-cost laser pointer, characterized by a low cost, ease of implementation, and high precision, is presented. Another important characteristic of this methodology is the synchronization of the moving load position and of the inclination and deflection measurement. The experimental test carried out on a real bridge demonstrates its usability for dynamic structures monitoring.

This paper covers: (1) the description of the methodology; (2) the hardware components (laser pointer, digital cameras, GNSS receiver, computer); (3) the software implemented (determination of laser footprint, time registration, inclination and displacement measurement); (4) the calibration procedures; (5) the in-field test; and (6) the discussion of results.

## 2. Materials and Methods 

### 2.1. The Methodology

The proposed method takes advantage of the laser pointers’ property to provide a steady pointing direction and produce a long-range, high-brightness visible imprint.

**Figure 1:**
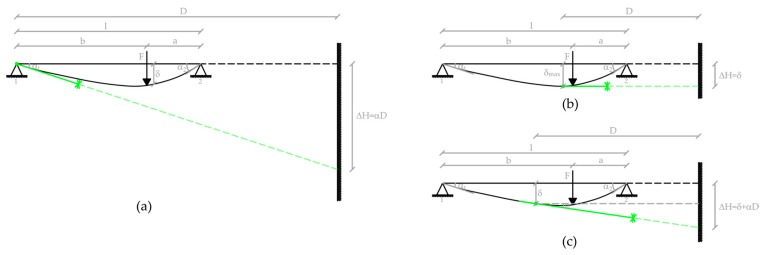
The displacement of the laser footprint in three cases: (**a**) laser fixed to a point subject only to inclination; (**b**) laser fixed to a point subject only to lowering; (**c**) laser fixed to a point with both inclination and lowering.

The footprint of a laser positioned at the intrados of a beam and projected on a plane target approximately orthogonal to the direction of the ray, will undergo a displacement ΔH due to two components: (a) the lowering or raising of the laser source and (b) the variation of the laser beam inclination. Both components are linked to the movements and inclinations of the structure to which the laser source is locked (Figure 1). 

The component (a) produces a shift δ of the laser footprint equal to the displacement of the laser source. The component (b) causes a displacement αD proportional to the distance D between the laser source and the target. It is therefore possible to greatly amplify this displacement by positioning the target at a convenient range; this allows one to obtain the tilt variation with remarkable precision.

Lowering and inclination vary depending on the type of structure and the point of application of the load [38]. For example, in the case of a point load applied to a simply supported uniform cross-section beam, which is the structural scheme used for most of the existing bridges, the displacement at a distance *x* from the support 1 is:

(1a) $$\delta_{x}=\frac{Fax\left(l^{2}-\;a^{2}-x^{2}\right)}{6lEI}\;\mathrm{for}\;0\;<\;x\;<\;b$$

(1b) $$\delta_{x}=\frac{Fb\left(1-x\right)\left[l^{2}-\;b^{2}-{\left(1-x\right)}^{2}\right]}{6lEI}\;\mathrm{for}\;b\;<\;x\;<\;1$$

For *b* < *x* < l, Equation (1a) can be still applied, considering *x* as the distance from support 2 and swapping *a* with *b*.

The maximum displacement is given by:

(2) $$\delta_{max}=\frac{Fa{\left(l^{2}-\;a^{2}\right)}^{3/2}}{9\sqrt{3}lEI}$$

This maximum deflection occurs at a distance x_1_ from the closest support, given by:

(3) $$x_{1}=\sqrt{\frac{l^{2}-a^{2}}{3}}$$

The slopes at the ends are:

(4) $$\alpha_{1}=\frac{Fa\left(l^{2}-\;a^{2}\right)}{6lEI}\;\alpha_{2}=\frac{Fab\left(2l-\;a\right)}{6lEI}$$

where:

*F* = Force acting on the beam
*l* = Length of the beam between the supports
*E* = Modulus of Elasticity
*I* = Area moment of Inertia of cross-section
*a* = Distance from the load to the support 2
*b* = Distance from the load to the support 1

The ratio between maximum deflection and the slope at end 1 is given by:

(5) $$\frac{\delta_{max}}{\alpha_{1}}=\frac{2\sqrt{\left(l^{2}-\;a^{2}\right)}}{3\sqrt{3}}$$

The ratio between the deflection at the distance *x* from the end 1 and the slope at end 1 is given by:

(6) $$\frac{\delta_{x}}{\alpha_{1}}=\frac{x\;\left(l^{2}-\;x^{2}-\;a^{2}\right)}{\left(l^{2}-\;a^{2}\right)}$$

By measuring the inclination at an extreme point where the laser pointer is fixed and by knowing the point of application of a load, it is therefore possible to obtain the lowering of the span at any point, by using Equation (6).

In the case of a different structural scheme, the relevant equations, easily findable in the civil engineering handbooks, should be used. The accuracy of the result depends on the correspondence between the as built and structural scheme of the design.

The measurement of the slope due to a load is obtained by the variation of the tangent to the elastic line, materialized by the laser beam projected by the pointer, fixed to the truss beam of the bridge, on a screen located at a suitable distance, in order to amplify the movement of the laser fingerprint and to get, therefore, a remarkable result accuracy (see Figure 1a). A video of the oscillations of the laser footprint is acquired; by analyzing the single frames, the variable position of the laser footprint centroid (ΔH in Figure 1a) gives information about the slope (angle α) changes and, consequently, about the dynamic deflections. The position of the dynamic load can be detected by a video and/or GNSS positioning. The synchronization of the acquisitions is performed by using GPS time.

A first test was carried out on a bridge, whose structure is a simply supported space frame girder.

### 2.2. The Hardware Components 

The hardware components are: (a) a laser pointer; (b) a digital camera for laser footprint video capturing and a camcorder for the video of the mobile load; (c) a GNSS receiver; and (d) a computer with a synchronized clock.

#### 2.2.1. The Laser Pointer

A SCITOWER SCT306-532 nm laser pointer was used. The main characteristics are resumed in Table 1.

The laser pointer was mounted on a Newport Research Corporation model 810 laser mount, provided with a strong magnetic base and two micrometric adjustment screws for two-axis positioning.

#### 2.2.2. The Digital Camera

The video of the laser footprint was acquired using a NIKON D610 camera with a 55 mm NIKKOR lens. The main characteristics are shown in Table 2.

As for the video of the mobile load (a truck), a Canon Legria HF R78 Full HD Handycam was used.

#### 2.2.3. The GNSS Receiver

The GNSS receiver is a Ublox NEO-M8T provided with a cheap patch antenna. The NEO-M8T is a timing receiver, but it can provide access to raw measurements on L1 (carrier-phase, pseudorange, Doppler) for all available GNSS constellations and augmentation systems. For our aims, the receiver was configured to track GPS and GLONASS.

#### 2.2.4. The Computer

A Dell XPS 13 9360 Notebook was used. The CPU is an Intel Core i7-7500U with a 2.7 GHz clock and 8 GB DDR SDRAM. The notebook is provided with a 256 GB SSD hard disk, a 13.3 inch Full HD display, and a graphic card Intel HD 620. The operating system is Windows 10 Pro. The synchronization with *time.windows.com* can be performed with an accuracy of 1 ms. Time format was set up in order to show hundredths of a second.

### 2.3. The Software Implemented

A program was developed in Matlab^®^ for the determination of the laser footprint centroid projected on a flat target. The program uses the results of the calibration of the digital camera, described afterwards. With regard to the mean scale of the frame, the Ground Sample Distance (GSD) is obtained at the beginning of the shoot, given that a millimeter paper glued to a rigid plastic tablet is used as the target. The millimeter paper allows one to obtain the GSD, theoretically different for each pixel, but it has to be considered that the target is fixed vertically and the camera optical axis is horizontal, so the scale of the image in the vertical direction is practically identical in all zones of the frame.

In order to obtain the position of the laser footprint centroid, for each frame, an intensity cut-off is preliminarily performed, which eliminates noises and the grid of the millimeter paper from the image. The centroid coordinates (row and column) are then calculated in pixels, through a weighted average: each pixel is assigned a weight equal to its intensity.

The row and the column of the centroid are:

(7) $$row=\frac{\sum_{i=1}^{n}\sum_{j=1}^{m}I_{i,j}i}{m\;n}$$

(8) $$col=\frac{\sum_{i=1}^{n}\sum_{j=1}^{m}I_{i,j}j}{m\;n}$$

where:

*row* = row coordinate of the centroid
*col* = column coordinate of the centroid
*n* = number of rows of the frame
*m* = number of columns of the frame
*I* = Intensity of the pixel

According to the literature regarding image processing, the coordinates of the centroid can be obtained with an accuracy of 0.1 pixels [39]. For our aims, this accuracy is redundant, since the pointing instability is greater than one pixel.

The coordinates of the centroid are then converted in mm, using the known GSD.

If the camera settings provide a very low ISO sensitivity and a small diaphragm aperture, you can get a better defined shape of the laser beam footprint and avoid image saturation in the center zone of the footprint. This allows a more accurate determination of the centroid.

Since the position of the centroid is given for each frame, it is possible to evaluate the displacement of a monitored point with a sampling rate equal to the 30 Hz frame rate of the camera. Thus, we obtain a graph of the centroid position as a function of time. Since the acquisitions of the video and of the moving load position are synchronized, the instantaneous displacement of the laser beam footprint is correlated to the position of the mobile load.

A module of the implemented software is devoted to the calculation of the deflection. In the first version, the laser pointer is considered fixed to an end of the bridge span; in this case, the deflection of the laser footprint is only due to the variation of the inclination of the laser beam, since the end of the span has no deflection. Inputs of the module are: (a) the distance from the laser pointer to the target; (b) the section inertia properties of the bridge in the case of non-uniform cross-section beams; and (c) the position of the mobile load acquired by the GNSS receiver.

Once the slope of the laser beam has been obtained, the deflection is computed in real time for a requested position, e.g. for the midspan, and for an uniform cross-section using Equation (6). The procedure is performed for each frame of the acquired video.

### 2.4. The Calibration Procedures

**Figure 2:**
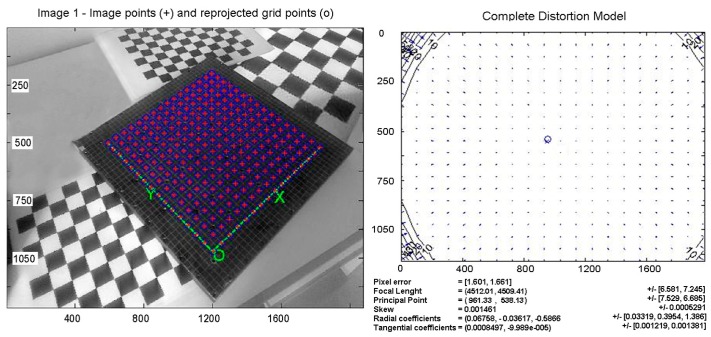
(**a**) The crosses of the calibration grid automatically recognized by the calibration software; (**b**) The distortion model of the calibrated camera. The coordinates and the results are in pixels.

For the calibration of the camera, an upgrade of a well-known procedure [40,41], developed using Matlab^®^, has been used. The procedure has been applied to the NIKON D610 camera with a 55 mm NIKKOR lens configured with a HD frame (1920 × 1080). A calibration plate with an accuracy of 0.1 mm has been used. Figure 2a shows the points of intersection automatically recognized by the software. If necessary, the operator can correct any errors or eliminate false positives identified by the automatic procedure. The main parameters of the tested camera are shown in Figure 2b. The results obtained are: focal length, principal point, skew, and radial and tangential distortion parameters.

After the camera calibration, to evaluate the accuracy of the centroid coordinates obtained by the aforementioned software, a lab test was carried out. The laser pointer was fixed to a linear motion system Impex HVP060 AM, characterized by a positioning precision of 0.03 mm.

**Figure 3:**
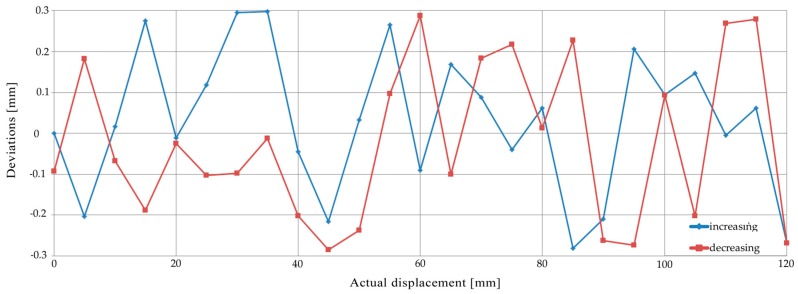
The deviations between the displacements obtained by the software and those from the displacements produced.

The beam was projected orthogonally on a flat target and the laser pointer was shifted by 120 mm, with 5 mm steps, by means of the drive unit. Shifting was applied many times back and forth, thus resulting in loop processes. In Figure 3, the deviations between the displacements of the centroid of the laser footprint obtained by the software and the ones produced by the linear motion system are shown for a loop. The horizontal axis represents the displacement of the laser beam; the blue line represents the deviations during the increasing displacements, whereas the red line represents the deviations during the decreasing ones. The maximum deviation is 0.3 mm, while the standard deviation is 0.13 mm.

To verify the laser pointer stability, the pointer and the target were positioned on the bridge to be monitored, in order to have the same layout to the one of the test to be carried out. Fifteen videos of five minutes were shot at an hour interval and, for each video, the oscillations of the laser fingerprint centroid were obtained. The short-term instability was one fifth of the declared pointing stability: in fact, a maximum oscillation of 5 pixels during each video was measured, corresponding to an angle of about 0.01 mrad, whereas the maximum difference measured in all videos was 14 pixels.

## 3. The Test

**Figure 4:**
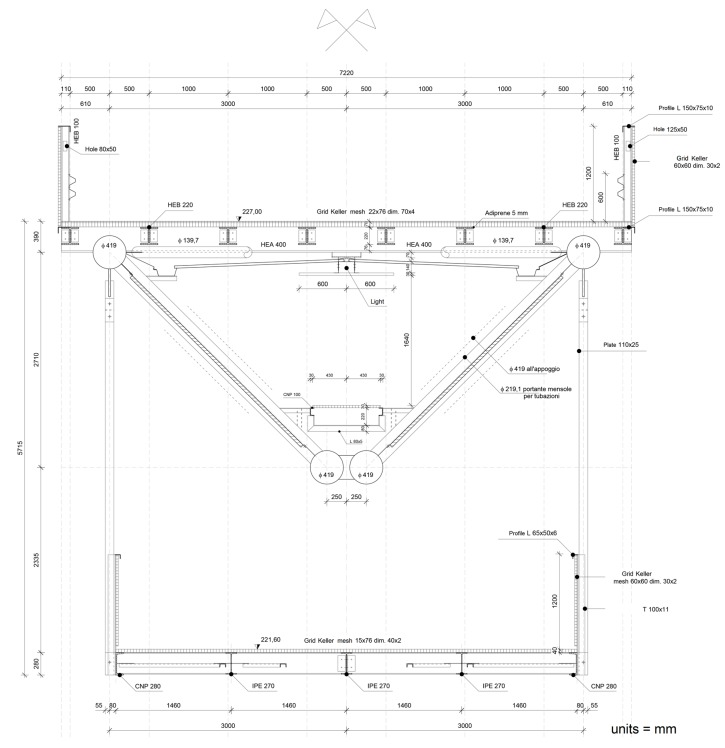
Cross section of the double-deck bridge at the University of Calabria.

The test was carried out on a bridge at the University of Calabria, Italy. The layout of the university is characterized by a South-North axis; all buildings are aligned to the sides of a central pathway built partly on double deck bridges: the upper deck can be used for vehicular traffic, while the lower one is reserved for pedestrians (Figure 4).

**Figure 5:**
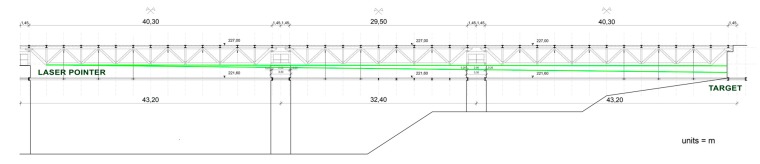
The layout of the test.

**Figure 6:**
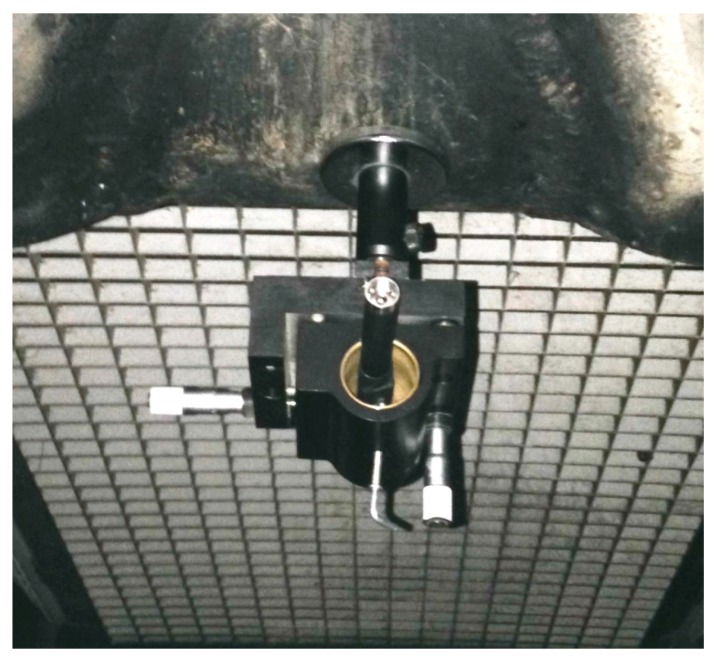
The laser pointer and the holder.

**Figure 7:**
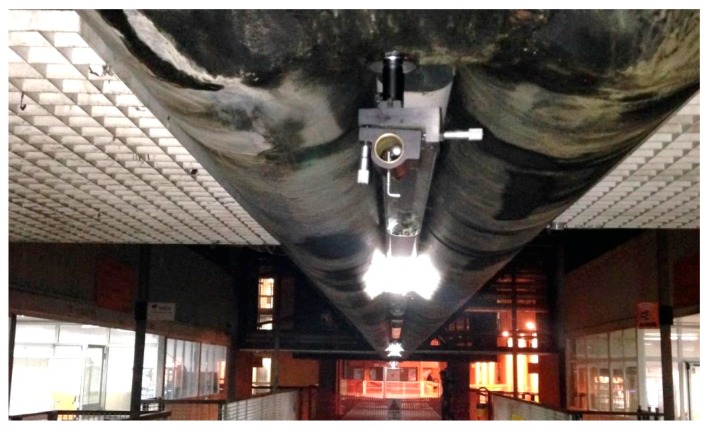
The pedestrian deck: the target is on the front wall.

The layout of the test is shown in Figure 5. The laser pointer is fixed to a tubular element of the space frame girder of the bridge, close to the end of the span (Figure 6 and Figure 7). The laser beam is projected onto an A4 size flat target, fixed to a vertical wall of the north terminal abutment. If the free space beneath the bridge is less than 30 cm, the target can be fixed to any fixed position, e.g., to a cap of a pile, or to a slope protection. To point exactly at the target, the pointer is mounted on a holder, usually used on optical tables, which allows precise horizontal and vertical movements. The holder is equipped with a strong magnetic base.

The NIKON 610 camera, used to obtain the footprint video, is positioned on a robust tripod, slightly lateral with respect to the laser beam path. The camera-target distance is chosen in order to obtain a Field of View (FoV) slightly larger than the dimensions of the target. Due to the camera-target distance and the lens focal length, the mean GSD is 0.25 mm. Taking into account the pointing stability of the laser pointer, a smaller GSD would be useless. Given that the distance from the laser pointer to the target is 115.70 m, we obtain a beam footprint diameter of 95 mm and a maximum theoretical pointing instability of 5.8 mm (23 pixels), taking into account the characteristics of Table 1. This suggests that the method can achieve good results even for shorter pointer to target distances.

The projection plate plane and the optical path are not angled. Actually, by using, e.g., a 30° angle between the laser beam and target plane like in [19], the movement of the laser spot centroid in the video image is amplified, and the measurement accuracy is theoretically increased, but this improvement is counterbalanced by the need to double the FoV and, consequently, the GSD.

The used technique allows us to determine the centroid of the footprint with an accuracy of less than one pixel, and thus the expected error in the measurements of the beam inclination is almost completely due to the laser pointer instability and can be conservatively evaluated as 0.05 mrad.

**Figure 8:**
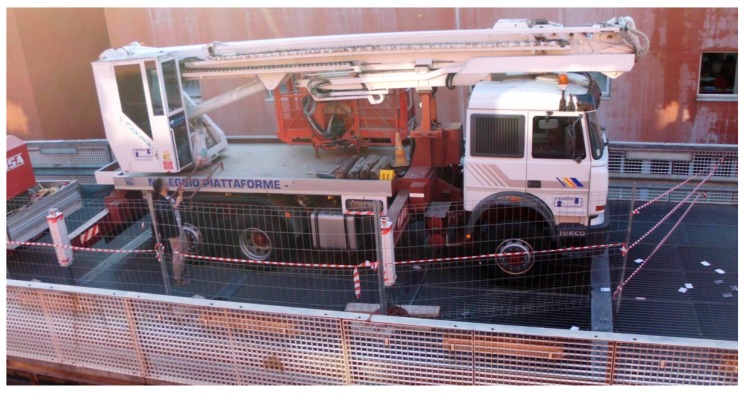
The truck elevator on the upper deck of the bridge.

The test was carried out during the movements of a truck elevator, which had been stationed for a few days and used for work on the façade of a building alongside the bridge (Figure 8). The patch antenna of the Ublox NEO-M8T receiver was positioned on the cab roof. The weight of the truck was about 260 kN. The video of the mobile load was shot with the camcorder when the truck left the bridge. Due to the limited space, the truck performed some forward and backward movements along the left span to reach the optimal alignment before the final reverse running at a speed of about 4 m/s.

With regard to time synchronization, the Nikon 610 camera is provided with a GP-1 unit, an accessory that can provide the Coordinated Universal Time (UTC). For synchronization of the camcorder, the display of the notebook, showing the GPS time rounded to hundredths of a second, was framed before and after the video shot. In this way, the video’s timing synchronization was obtained with an approximation equal to its frame rate of 30 fps.

**Figure 9:**
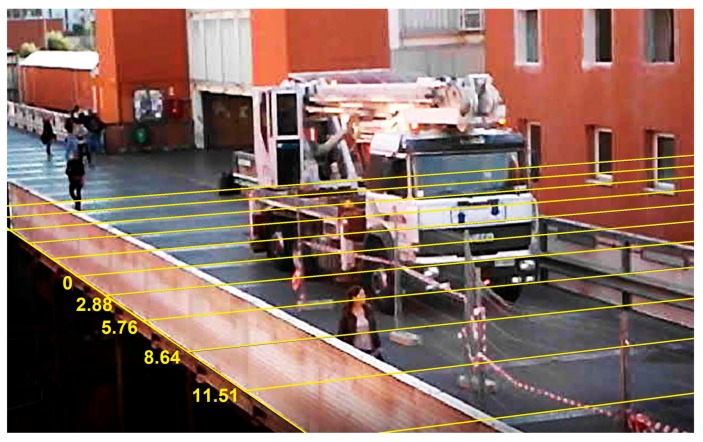
A frame of the camcorder video with the truck leaving the bridge. The transverse beams on the deck are used to obtain the position in the direction parallel to the longitudinal axis of the bridge.

A frame of the video is shown in Figure 9. The image shows the truck during the backward running. The transverse beams of the upper deck, positioned every three meters, allow one to determine the position of the wheels in the longitudinal direction. The origin of abscissae (positive in the North direction) is fixed at the south end of the span.

The accurate abscissae of the truck were obtained by cinematic differential positioning. The Ublox GNSS receiver was set to acquire data with a 5 Hz sampling rate, while the fixed GNSS station at the University of Calabria was used as the base. Furthermore, two points on transverse beams of the upper deck were previously surveyed, in order to perform a coordinate transformation and obtain the abscissae in the local reference system.

## 4. Results and Discussion

**Figure 10:**
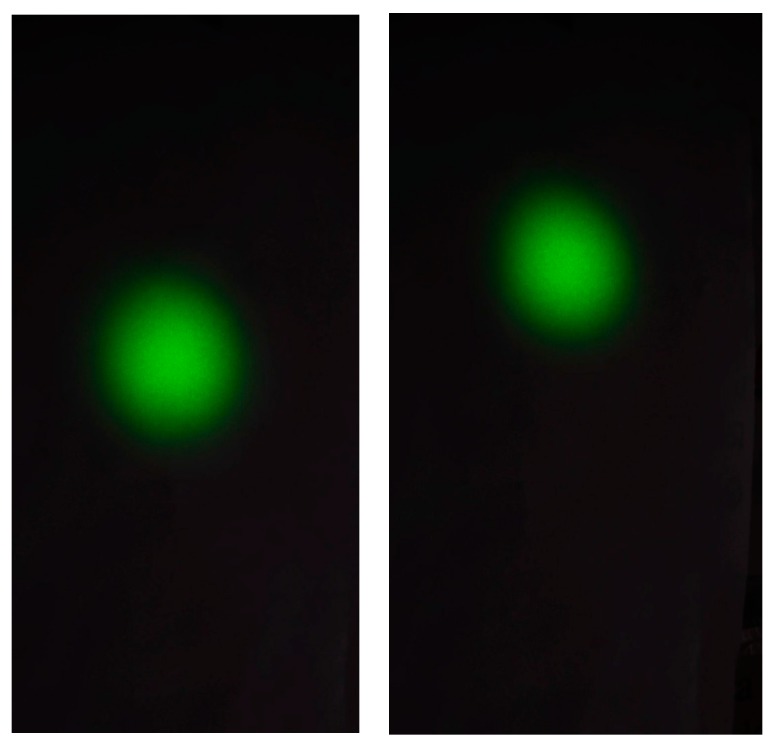
Two frames acquired at the beginning and at the end of the test: after the truck left the bridge, the footprint is higher.

In Figure 10, we can observe two frames obtained at the beginning and at the end of the test. The ISO sensitivity and the aperture were chosen in order to obtain a radiometric cut off, thus achieving two goals: a better defined shape of the laser beam footprint was obtained and the saturation of the image in the center zone of the footprint was avoided. This allows a more accurate determination of the centroid. The frames were processed using the code in Matlab^®^ previously described and the dynamic position of the centroids in pixel coordinates (rows, columns) was obtained.

**Figure 11:**
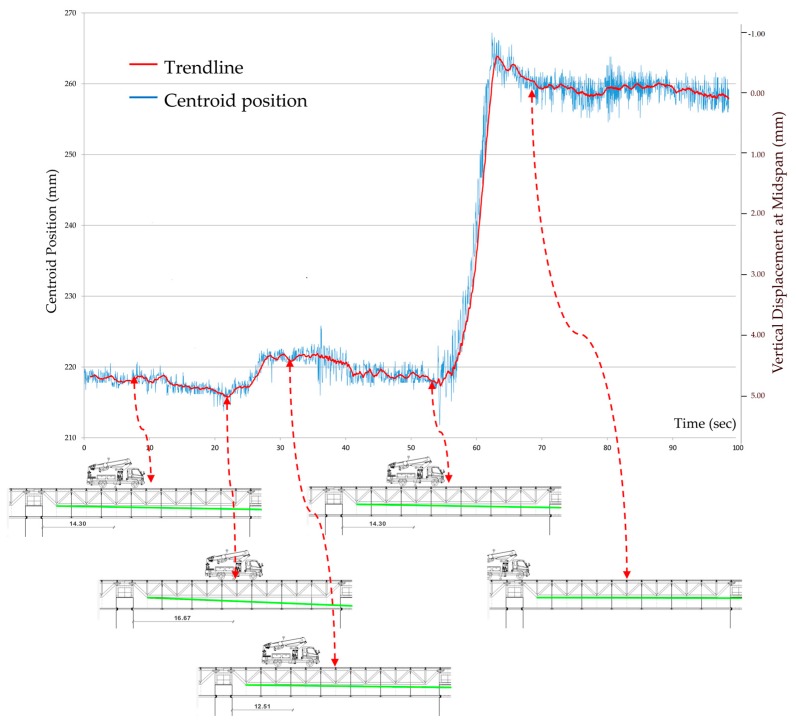
The vertical position of the centroid of the footprint during the test.

In Figure 11, the height of the centroid during the test is shown. In red, a trend line (30-sample moving average) is drawn. The origin of ordinates is at the bottom of the frame and the values have been transformed from pixels into mm, while the scale of the frames has been obtained using the known GSD. Abscissae are in seconds. The y axis on the right gives the vertical displacements at the midspan, calculated using Equation (6). The origin has been fixed in correspondence of the position assumed by the centroid after the end of the test; we can consider this situation as a zero deflection condition since the load applied during the test does not cause plastic deformations.

It is possible to observe that the pointing stability was less than 0.05 mrad (corresponding to 5.8 mm for the pointer-target distance equal to 115.70 m).

From a qualitative point of view, we can observe that the forward—backward movements of the truck are clearly reflected in the movements of the laser beam. Furthermore, some damped oscillations are recognizable after the truck left the bridge.

As regards the truck position, the abscissae (previously defined and shown in Figure 9) obtained using the GNSS positioning were used for the elaborations. A comparison between the abscissae obtained by the camcorder video and by the GNSS solution showed a maximum deviation of 0.3 m.

The span of the bridge is 40.30 m, while the barycenter of the truck, at the beginning of the test, is located 14.30 m from the bearing.

After 55 s from the beginning of the video, a sudden variation is evident, equal to about 40 mm, corresponding to an inclination change of 0.346 mrad. In this period, the truck left the bridge at a constant speed, which is reflected in the parabolic decrease of the vertical displacement at the midspan. Given that the laser is positioned very close to the bearing and the section inertia properties of the truss beam are constant, the estimation of the maximum deflection can be made using Equation (5). The variation of the maximum truss beam deflection obtained this way is 5.0 mm. The variation of the deflection in the midspan, obtained by using Equation (6), is 4.9 mm.

A more accurate evaluation was performed using a FEM code, written at the University of Calabria [42]. The obtained deflection was 4.8 mm.

A precise measurement was conducted using a Leica 1201+ total station. The axis of a bolt of a steel connection in the midspan was used as a target and its position was surveyed before and after the test. The measured variation of the truss beam deflection was 4.8 mm, 2% less than the result obtained with the proposed methodology.

The use of the positions obtained by the camcorder video and by the GNSS solution gave comparable results, with differences of less than 5%. Both techniques have pros and cons. GNSS allows one to obtain more accurate positioning along with a simple and accurate time synchronization, but a receiver must be installed on the vehicle. The video gives less accurate positioning, and implies more computer processing both for images and time synchronization, but it can also be used for non-instrumented vehicles. For official load tests, the first technique is the most suitable; in fact, in order to obtain the requested precision of positioning, the vehicles used as mobile loads can be easily provided with a GNSS receiver. For the monitoring of a bridge under normal conditions, instead, the second technique is the only one which is currently usable.

## 5. Conclusions

In light of the results of the test carried out on a real case, we can conclude that the method proposed allows one to obtain the displacement at an arbitrary point of a bridge using the load position measured by GNSS or a camcorder video and rotational angle at an end measured synchronously by laser projection-sensing technology. The rotations are obtained with the precision required for load tests and monitoring. The low cost of the components and the ease of configuration make the method a suitable alternative to the traditional methods. Its reliability has been demonstrated both from a qualitative and quantitative point of view. The forward—backward movements of the truck used for the experimental test are clearly reflected in the movements of the laser beam. For the bridges with a simply supported uniform cross-section beam, which represent most of the existing bridges, the displacements can be estimated with a good accuracy. In our test, the deviation between the beam deflection obtained with the described method and the one measured by a high-end total station was about 2%.

Along with the precision obtained, a noticeable goal is the synchronization of the acquisitions, which allows one to ascertain the instantaneous position of the mobile load and the deflection.

In the near future, the use of a camera with high frame rate is foreseen, in order to demonstrate the usefulness of the method for the control of the bridge’s natural frequencies.

## Figures and Tables

**Table 1:** Characteristics of the laser pointer.

Feature	Value
Wavelength	532 ± 0.1 nm (Green)
Beam diameter	2.0 mm
Beam divergence	0.8 mrad
Power	100 mW (Gaussian Beam)
Pointing stability	<0.05 mrad
Beam spot roundness	≧90%
Laser distance	~500 m
Warm-up time	≦1 min
Lifetime	≧8000 H

**Table 2:** Characteristics of the NIKON D610 digital camera.

Feature	Value
Type	Single-lens reflex digital camera
Effective pixels	24.3 million
Image sensor	Nikon FX format 35.9 × 24.0 mm—DX format 24 × 16 mm
File format	NEF (RAW), JPEG, NEF (RAW) + JPEG
Lens	NIKKOR 18–55 mm f/3.5–5.6 G VR
Shutter	Electronically-controlled vertical-travel focal-plane shutter
ISO sensitivity	ISO 100 to 6400 in steps of 1/3 or 1/2 EV
HD frame and frame rate	1920 × 1080 pixels; 30 p (progressive), 25 p, 24 p
